# The future of preconception care in the United States: multigenerational impact on reproductive outcomes

**DOI:** 10.1080/03009734.2016.1206152

**Published:** 2016-07-15

**Authors:** Michelle St. Fleur, Karla Damus, Brian Jack

**Affiliations:** Department of Family Medicine, Boston University School of Medicine/Boston Medical Center, Boston, MA, USA

**Keywords:** Epigenetics, health disparities, perinatal outcomes, preconception care, preconception health

## Abstract

The future of preconception care will require an innovative multigenerational approach to health promotion for women and men to achieve optimal reproductive health outcomes. In this paper we provide a summary of historical trends in perinatal interventions in the United States that have effectively reduced adverse perinatal outcomes but have not improved disparities among ethnic/racial groups. We describe evidence pointing to an enhanced preconception care paradigm that spans the time periods before, during, and between pregnancies and across generations for all women and men. We describe how the weathering, Barker, and life course theories point to stress and non-chromosomal inheritance as key mediators in racial disparities. Finally, we provide evidence that indicates that humans exposed to toxic stress can be impacted in future generations and that these phenomena are potentially related to epigenetic inheritance, resulting in perinatal disparities. We believe that this expanded view will define preconception care as a critical area for research in the years ahead.

## Introduction

Given the myriad of factors that influence preconception health (1), the future of preconception care will depend on how well the progress made so far can be leveraged and expanded. It will also rely on how new scientific evidence can be integrated into the content, risk assessment, and mitigation of preconception risks. The strategies and resources used to facilitate the clinical and public health integration and dissemination of preconception care, including the role of group care, the medical home, workplace and school-based health promotion programs, and home visitation, will also impact future progress. Although in the past decade preconception health (PCH) and preconception care (PCC) have gained momentum, the number and types of effective evidence-based interventions are limited and have remained relatively stable, and, as for all maternal child health (MCH) interventions, there is wide global variability, reflecting regional and national specific challenges and resources. Some of these key influencing factors include: the priorities placed on women’s, men’s, and families’ health, women’s reproductive rights, political will, access to and support for preventive health services, raising public health awareness and education, health care provider training, reimbursement of services, developing, implementing, and disseminating evidence-based interventions, baseline perinatal outcomes, and population health status. There is also strong support of other key variables that impact PCH/PCC with equal if not greater international variability, such as the importance and influence of the social determinants of health, the role of childhood adverse events, toxic stress, and epigenetics. Therefore, discussions and predictions about the future of preconception care must integrate all of these issues into a cohesive context, reflecting how each of the factors might influence the future of PCC.

In this paper, after a brief summary of historical trends in seminal perinatal outcomes in the United States (USA), we will present an overview of some of these factors that underscore the need for PCC from the lens of the persistent ethnic/racial disparities and describe how an innovative epigenetic transgenerational approach can portend the future of preconception care.

## Historical trends and ethnic/racial disparities in perinatal outcomes in the USA

From 1940 to 2005 the overall infant, neonatal, and post-neonatal annual mortality rates in the USA declined substantially (2). The infant (age less than one year) mortality rate (IMR) decreased by 85%, from 47 per 1,000 live births in 1940 to 6.9 in 2005; the neonatal (age less than 28 days) rate decreased by 84%, from 28.8 to 4.54; and the post-neonatal (age greater than 28 days but less than one year) rate decreased by 87%, from 18.3 to 2.34 (3). After a plateauing of IMRs in the USA from 2005 to 2010, rates began to fall again, reaching a historical low of 6.0 in 2013 (4). But despite this progress, the USA continues to have the highest IMR of all Organization for Economic Co-operation and Development (OECD) countries (5). Furthermore, ethnic/racial disparities in perinatal outcomes continue to persist in the USA. For example, in 2013 the majority (76.1%) of the nearly 4 million births in the USA were white and 16.1% were black (6), and the 2013 IMR for black infants was 11.1 per 1,000 births compared to 5.1 for white infants. Therefore, black babies were still twice as likely to die in their first year of life compared to white babies. In addition, black women were also almost twice as likely to deliver a low-birth-weight (LBW) baby (≤2,500 g) and three times as likely to deliver a very-low-birth-weight baby (<1,500 grams) than white women (7). Socioeconomic factors (8), genetics, health behaviors, experience of stressful life events (9), and toxic stress (10) have all been studied, but despite decades of programs and research, the factors that contribute to this disparity and effective interventions to promote equity remain poorly understood (11). With LBW and short-term gestation being the leading cause of mortality for black infants (3,12), we will not optimize maternal child health (MCH) outcomes until we reduce these disparities (13,14).

## Strategies used to improve maternal childhealth outcomes

Maternal child health (MCH) research over the past 40 years has focused on improving hospital care, prenatal care, and, more recently, preconception care. In an effort to improve MCH outcomes, research has continued to go back in time to address the root causes. Intrapartum, antenatal, and preconception care have contributed to improved outcomes, but they have been shown only minimally to impact disparities in IMR and LBW. Between the 1960s and 1990s, the focus was on improving labor and delivery. This included hospital deliveries, medicalization of birth, electronic fetal monitoring, and ultrasound. Some outcomes improved, some worsened, but the disparities remained (15).

From the 1980s to 1990s the focus then shifted to antenatal care. In 1968, Gortmaker et al. examined all births in New York City and found a strong association between preterm deliveries and inadequate prenatal care (16). Since then, the association between inadequate antenatal care and low gestation weight is well documented (17–19). As a result, increased efforts were made to improve access to and quality of prenatal care. However, intervention studies did not show improvements in disparities.

With preterm premature rupture of membranes (PPROM) identified as the leading cause of preterm births (PTB) (<37 weeks of gestation) in black women, and intrauterine infections often being a precursor to PPROM, treating infections such as asymptomatic bacteriuria, pneumonia, chlamydia, and bacterial vaginosis should have led to reductions in black–white differences in PTB. Yet, the treatment of infections during prenatal care has also had little impact on disparities in the USA (12,20–22).

Starting in the 1990s to the present there has been a focus on understanding infant mortality disparities through preconception care (PCC). Preconception care is defined as a set of interventions that aim to identify and modify biomedical, behavioral, and social risks to the woman’s health or pregnancy outcome through prevention and management prior to a woman becoming pregnant. Strong evidence for some risks include folic acid deficiency, type 1 diabetes, smoking, phenylketonuria, and many others (23). However, to date there is no study showing that the ‘package’ of PCC services improves MCH outcomes. Data exist that we can identify PCC risks, but there are no data that this impacts MCH outcomes or disparities (2). It is possible that intrapartum, antepartum, and preconception services are necessary but not sufficient to improve perinatal outcomes among black women in the USA.

More recent and emerging data show that factors impacting prior generations can possibly explain perinatal outcome disparities. Since 2005, there has been a focus on better understanding risk factors that impacted previous generations and potential transgenerational effects. Explanatory mechanisms have included the life course perspective (9), Barker hypothesis (24), adverse childhood events (25), toxic stress (10), healthy immigrant effect (26), impact of stress and societal racism (27,28), weathering hypothesis (29), and epigenetics (30) as possible mechanisms.

## Life course theory and the Barker hypothesis

The life course perspective by Lu et al. (31) posits that disparities in birth outcomes are the consequences of differential developmental trajectories that are set by early life experiences and cumulative allostatic load over the life course (31). It is especially important in MCH, where one developmental stage often gets disconnected from another. In perinatal health, the focus is so much on events occurring in the 40 weeks of pregnancy that it is forgotten that there are a great deal of life course influences on perinatal outcomes which impact health and illness potential throughout life (31). For example, in explaining the black–white gap in infant mortality, for decades there was a search for maternal risk factors during pregnancy rather than looking at the mothers’ cumulative life course experiences. The danger of focusing solely on risk factors during pregnancy is that since it does not adequately explain the disparities it can misguide public health interventions and policies.

The life course perspective assumes that accumulating stress throughout life somehow results in worsening MCH outcomes. But evidence is emerging that lifetime stress does not predict LBW or PTB. Among 33,452 women in the Pregnancy Risk Assessment Monitoring System (PRAMS) data set, black women reported the highest number of stressful events 12 months before delivery when compared to white women. These stressful events included emotional stress, financial stress, partner-related stress, and traumatic stress. But, in stepwise regression, stressful events were not associated with PTB in that generation X (9). Is it possible that the stressful events necessary to impact MCH outcomes must accumulate over a longer period of time—perhaps even over a generation or more?

The ‘Barker hypothesis’, which demonstrates that much of an adult’s health is programmed during his or her experience as a fetus and in early childhood, provides additional insights into adverse and disparate perinatal outcomes. A nationally representative sample in the USA found that children born <2,500 g were at increased risk (OR of 2.16, *P* < 0.01) for stroke, myocardial infarction, heart disease, and PTB by 50 years of age (24). Stroke mortality rates among adults in England and Wales are higher among people with lower birth weights. The mothers of these low-birth-weight babies were typically poor, malnourished, had poor overall health, and were generally socially disadvantaged. The odds of stroke more than doubled for those with birth weights <2,500 g compared with those weighing 4,000 g (32). Therefore it seems that your mother’s experience, not only yours, leads to adult disease and PTB (32). Since a stressful intrauterine environment or being delivered prematurely does not change the genetic code, by what mechanism does this lead to adult disease and subsequent preterm birth?

For two decades we thought if we could get women universal access to good-quality prenatal care, then we could influence maternal child health disparities. It is recognized now that to expect prenatal care, in less than nine months, to reverse all the cumulative disadvantages and inequities over the life course of the woman is expecting too much of prenatal care, particularly since many predictive adverse exposures begin during childhood.

## Adverse childhood events and toxic stress

Adverse childhood experiences (ACEs) are traumatic events (such as physical, emotional, or sexual abuse, parental divorce, or the incarceration of a parent or guardian), occurring before the age of 18, that the person remembers as an adult. ACEs are common, and they have long-term associations with adult health risk behaviors, health status, and diseases. The ACE Study was the largest study that examined the association between adverse childhood events and leading causes of death in 18,000 adults (25). These events included seven categories of adverse childhood experiences: psychological, physical, or sexual abuse; violence against mother; or living with household members who were substance abusers, mentally ill or suicidal, or ever imprisoned. The number of categories of these adverse childhood experiences was then compared to measures of adult risk behavior, health status, and disease. They found that more than half of respondents reported at least one exposure, and one-fourth of respondents reported ≥2 categories of childhood exposures. The number of categories of adverse childhood exposures had a positive graded relationship with the leading causes of death in adults, including ischemic heart disease, cancer, chronic lung disease, skeletal fractures, and liver disease.

Furthermore, these childhood exposures compromise the cognitive and emotional development of children as well as their capacity, as adults, to care for the next generation. Prolonged activation of the stress response systems in the absence of protective relationships can lead to toxic stress. Over time this leads to elevated cortisol levels and decreases in synapses between the cortex and limbic system (10). This stress can have intergenerational effects, with emerging research demonstrating changes at a genetic level. This long-term impact of early adversity on parenting perpetuates a cycle of intergenerational transmission of trauma and health risks.

There is also evidence that increasing societal stress can impact MCH outcomes. One example is the healthy immigrant effect, which is the phenomenon by which immigrants experience more positive health outcomes (including preterm birth) than the native-born population in developed countries (26). Is it possible that native-born foreign populations have more stressful lives than immigrants? If so, it is possible that life stress is related to adverse reproductive outcomes (26). Other theories have hypothesized the role of preconceptional stress and its contribution to the high rates of preterm birth experienced by black women (33). One such hypothesis includes the weathering hypothesis; this theory holds that social inequalities leads to an earlier and disproportional decline in the health status of minorities, resulting in widening health disparities between blacks and whites (34). In a study by Geronimus et al., black women living in low-income neighborhoods in Michigan were found to have a 3-fold increase of preterm births and a 4-fold increase in very preterm births (<32 wks) between the ages of 15 and 34 years when compared to white singleton births (34). It appears that either directly or indirectly, through interactions with host genes, stress is an important risk factor for adverse birth outcomes.

## Epigenetics and multigenerational effects on maternal child health

There is now evidence that the ‘epigenome’ or pattern of DNA methylation that is laid down during early fetal life might provide a mechanism to explain how intrauterine stress could impact adult health status and the propensity for PTB in the next generation. This variation occurs in the absence of alteration of the gene sequence. The degree of methylation affects how easily the enzymes that transcribe the genes can do their job. Therefore, the epigenome learns from its experiences. Epigenetic tags act as a kind of cellular memory.

While there is rapidly accumulating evidence in animal models that epigenetics can influence the health of future generations, there is now emerging evidence that this could be the case on humans as well. The 1944–1945 Dutch famine (35,36) provides empirical support for the hypothesis that early-life environmental conditions can cause epigenetic changes in humans that persist throughout life. Individuals who were prenatally exposed to famine during the Dutch hunger winter in 1944–45 had, six decades later, less DNA methylation of the imprinted *IGF2* gene compared with their unexposed, same-sex siblings. The association was specific for periconceptional exposure, reinforcing that very early mammalian development is a crucial period for establishing and maintaining epigenetic marks. These data are the first to contribute empirical support for the hypothesis that early-life environmental conditions can cause epigenetic changes in humans that persist throughout life. The periconceptual environment may represent a window of vulnerability during which differential methylation could occur in response to severe environmental stress (35).

Additional human data that lend support to the theory that stressful events can impact birth outcomes in subsequent generations are provided in studies by Collins known as the Grandmother Epidemiologic data set (37) that links three generations of African Americans in Illinois. Rates of LBW were associated with worsening maternal grandmothers’ residential environments during her pregnancy with her daughter, who years later delivered a LBW neonate. This association was independent of the living conditions of the daughter during her pregnancy with the infant with low birth weight. This transgenerational effect supports the notion that epigenetic mechanisms are likely to play a role in the pathophysiology of preterm labor (37).

In a pregnant mother, three generations (the mother, her daughter, and eggs of the daughter) are directly exposed to the same environmental conditions at the same time. In this way, three generations are simultaneously exposed to the same environmental stressors such as diet, toxins, and stress, among others (37). In order to provide a convincing case for epigenetic inheritance, an epigenetic change must be observed in the fourth generation.

Data from The Newborn Epigenetics Study cohort in North Carolina measured stress in 537 women at 12 weeks’ gestation using the Perceived Stress Scale. DNA methylation at differentially methylated regions was measured from peripheral and cord blood. Maternal stress was not associated with PTB. However, elevated stress was associated with higher infant DNA methylation (2.8% difference, *P* < 0.01). This provides evidence that maternal stress may be associated with epigenetic changes that are passed on to their daughters (38).

Another study showed that severe maternal depressed mood was associated with a 3-fold increase in the risk of LBW and that DNA methylation levels of the offspring differed significantly by maternal depressed mood, measured by using the Centers for Epidemiologic Studies Depression Scale (39), providing further human evidence that epigenetic mechanisms could be involved.

If some of the social determinants of health (socioeconomic status, ethnicity/race, education, violence, experiences of racism, health behaviors such as dietary practices, and environmental exposures) are found to affect the degree of DNA methylation and differences in phenotype, then one of the mysteries of the gene–environment interactions could be solved.

## Overall implications for the future ofpreconception care

To achieve optimal pregnancy outcomes in the future, which will translate to better health throughout life, enhanced preconception care that spans the time periods before, during, and between pregnancies and across generations for all women and men is needed. This expanded view of PCC requires a paradigm shift to an epigenetic translational approach and strategies that integrate evidence-based science about biomedical risks, toxic stress, social determinants of health, combined with an informed public and providers, adequate health care services, and the effective use of tested technologies for dissemination. Such a model should also help to promote equity in outcomes since the life course, Barker, and weathering theories all point to stress and non-chromosomal inheritance as key mediators in ethnic/racial disparities. Epigenetic changes have been shown in humans exposed to toxic stress that can manifest changes that impact future generations, so that a grandmother’s stress can result in a preterm birth of her grandchild. Finally, multigenerational data sets that include bio-behavioral risk factors, measures of environmental and social stress, and reproductive outcomes are needed to analyze the complex relationships that must be studied to inform the content and the effective evidence-based PCC interventions that are yet to be identified.
